# Association of bleb formation with peri-aneurysmal contact in unruptured intracranial aneurysms

**DOI:** 10.1038/s41598-022-10064-8

**Published:** 2022-04-12

**Authors:** Toru Satoh, Takanobu Yagi, Yoichi Sawada, Kenji Sugiu, Yu Sato, Isao Date

**Affiliations:** 1 Department of Neurological Surgery, Ryofukai Satoh Neurosurgical Hospital, 5-23-23 Matsunaga, Fukuyama, Hiroshima, 729-0104 Japan; 2 Center for Advanced Biomedical Sciences, Waseda University, 2-2 Wakamatsucho, Shinjukuku, Tokyo, 162-8480 Japan; 3 Department of Contemporary Welfare, Faculty of Health and Welfare, Okayama Prefectural University, Okayama, Japan; 4 Department of Neurological Surgery, Okayama University Graduate School of Medicine, Okayama, Japan

**Keywords:** Neuroscience, Neurology, Nanoscience and technology

## Abstract

The mechanism of bleb formation in unruptured intracranial aneurysms (UIAs) remains unclear. This study aimed to investigate the association between peri-aneurysmal contact (PAC) and bleb formation. Forty-five aneurysms were classified depending on the presence of blebs and PAC using computed tomographic angiography and magnetic resonance imaging. Aneurysmal hemodynamics were assessed using computational fluid dynamics. The independent variables associated with bleb formation were statistically assessed. Fourteen aneurysms (31.1%) had blebs, all of which were located at the site of PAC (group A). Thirty-one aneurysms (68.9%) had no bleb, of which 13 had a PAC (group B) and 18 had no PAC (group C). PAC was the only independent variable associated with bleb formation (*p* < 0.05). Aneurysmal volumes were significantly higher in group A, followed by groups B and C in series. Aneurysmal wall shear stress (WSS) tended to be lowest in group A, followed by groups B and C in series. The maximum WSS at the blebs was only 17% of the maximum WSS at the aneurysmal domes. This study demonstrated that bleb formation in UIAs was associated with the establishment of PAC during their growth, which may have more detrimental effects on bleb formation than hemodynamics.

## Introduction

An intracranial aneurysm is a disease state of the cerebral artery, which often bulges outward to form a saccular shape. The prevalence of unruptured intracranial aneurysms (UIAs) is 3–5% in the adult population^1,2^. The annual rate of aneurysmal rupture is approximately 1%^3,4^, resulting in subarachnoid hemorrhage with a high mortality rate of 45%^5^. UIAs at a higher risk of rupture are treated with surgical or endovascular therapies. The rupture risk is often associated with their morphology, such as size, aspect ratio, and bleb formation^3,4,6^. A bleb is a secondary bulge of the aneurysmal wall, which may be caused by a focal increase in wall vulnerability, but the mechanism of bleb formation in UIAs remains unclear.

Peri-aneurysmal contact (PAC) and intra-aneurysmal hemodynamics may be associated with bleb formation. Several studies that compared ruptured and unruptured aneurysms demonstrated that ruptured aneurysms were larger and more irregular with more constraints due to the presence of PAC^7–10^. Hemodynamic parameters associated with bleb formation have been explored in UIAs, resulting in two schools of thought: one supported strong inflow jets and high wall shear stress (WSS)^11–13^ and the other supported low WSS and a high shear stress gradient^14,15^.

The above studies required further clarification on the association of bleb formation with PAC, especially in UIAs, since the morphology of aneurysms changes more or less after rupture^16^. In addition, previous studies have not compared the effects of PAC and hemodynamics. Thus, the present study aimed to investigate the association between PAC and bleb formation in UIAs, including effects due to aneurysmal morphology and hemodynamics.

## Methods

### Patient population

This retrospective study was approved by the Institutional Review Board (IRB) of Satoh Neurosurgical Hospital, and the protocols used in the study were approved by the Committee of Human Subjects Protection of the Satoh Neurosurgical Hospital, Hiroshima, Japan. All methods were performed in accordance with the relevant guidelines and regulations. Informed consent was obtained from all patients to use their clinical data. Forty-nine UIAs from 49 consecutive patients between September 2017 and August 2019 were analyzed. Four patients were excluded due to poor imaging, resulting in a total of 45 aneurysms enrolled in this study (13 men and 32 women). The mean age of patients was 65.07 ± 9.57 years (mean ± SD), with a range of 39 to 78 years. The locations of the aneurysms were as follows: 19 in the middle cerebral artery (MCA) (42.2%), 15 in the anterior communicating artery (AComA) (33.3%), five in the internal carotid-posterior communicating artery (IC-PC) (11.1%), four in the tip of the basilar artery (BA-tip) (8.9%), and two in the anterior choroidal artery (AChorA) (4.4%). Outcomes for these aneurysms included clipping (7 cases), coiling (2 cases), follow-up (4 cases), and subarachnoid hemorrhage (1 case) (Table 1). Treated aneurysms (10 cases) were analyzed using image datasets taken less than three months before treatment, and untreated aneurysms (35 cases) were analyzed based on the initial diagnosis.

**Table 1:** Clinical features.

Category	
Nr	45
Male/female	13/32
Age (y.o.)	65.07 ± 9.57
Location (Nr.)	45
MCA	19
AComA	15
IC-PC	5
BA-tip	4
AChorA	2
Outcome (Nr.)	Clipped: 7
	Coiled: 2
	SAH: 1
	Follow up:35

AChorA, anterior choroidal artery; AComA, anterior communicating artery; BA-tip, tip of the basilar artery; IC-PC, internal carotid-posterior communicating artery; MCA, middle cerebral artery; SAH, subarachnoid hemorrhage.

### CTA

Three-dimensional computed tomographic angiography (CTA) was performed using a multidetector CT system (Activion-16; Canon Medical Systems). The Real-Prep scan mode was used with 100 ml of contrast agent (Iomeron, iodine concentration 350 mg/mL; Eizai Pharmaceutical) injected at 3 mL/s into the antecubital vein. The imaging parameters included a 16-cm field of view, 512 × 512 matrix, section thickness of 0.5 mm, scanning time of 8 s, and a total of 201 images. The original volume data were interpolated into a matrix of 1024 × 1024 pixels and a thickness of 0.3 mm using a workstation (Ziostation-2, Ziosoft/AMIN, Tokyo).

### MRI

Magnetic resonance cisternography (MRC), in combination with magnetic resonance angiography (MRA), was performed for each patient on a 3 T unit (Signa Pioneer; GE Healthcare, Milwaukee, WI). A heavily T2-weighted 3D fast spin-echo sequence was used for the MRC with the following parameters: TR/TE, 1900/100 ms; field of view, 180 × 180 mm; matrix, 356 × 256; section thickness, 1.2 mm; NEX,1; bandwidth, 31.2 kHz; echo-train length, 128; acquisition time, 6 min. Sixty axial images were acquired. The volume data were interpolated into 1024 × 1024 pixels matrix and a thickness of 0.3 mm using the workstation.

### CFD

Computational fluid dynamics (CFD) was performed using a commercial CFD package (Hemoscope v1.4, EBM Corp., Tokyo). Blood was assumed to be an incompressible Newtonian fluid with a density of ρ = 1050 kg/m^3^ and viscosity μ = 0.004 Pa·s. Vascular geometries were reconstructed from CTA datasets and filled with unstructured hexahedral cells mainly. The mesh size was 0.25 mm in far-wall regions. The near-wall mesh consisted of three layers, with a width of 0.125 mm and a height of 0.05 mm at the wall-nearest mesh. Unsteady pulsatile simulations were carried out as a time step was adjusted to keep the Courant number less than one. As for boundary conditions, a time-averaged flow rate *Q* was first estimated using a formula *Q* = (τπ⁄32μ) *D*^3^, where τ is a magnitude of WSS (τ = 1.5 Pa) ^17^ and *D* is the diameter of the inlet artery. The distribution of flow rate at vascular bifurcations was set to be a ratio of the cubic diameters of the branches. A physiological pulsatile waveform was imposed on the steady flow rate^18^.

### Data extraction

#### Aneurysmal morphology

Based on the CTA datasets, a surface mesh of the vascular geometries was obtained. Aneurysmal shape indices (neck width, dome depth, aspect ratio, dome surface area, and dome volume) was computed using a surface mesh (Hemoscope v1.4, EBM Corp., Tokyo). The neck was defined as a plane whose location was determined by neurosurgeons. The neck width was the diameter of a circle with an area equal to that of the neck. Dome depth was defined as the length from the neck center perpendicularly towards the dome.

#### Identification of blebs and PAC

Blebs were determined by neurosurgeons as secondary focal bulges distinguishable from an aneurysmal dome based on 3D-CTA datasets. The peri-aneurysmal environment was visualized using 3D multifusion CTA and MRC images^9^. The presence of PAC was judged based on whether the aneurysms overlapped with the surrounding structures. If aneurysms harbored blebs, the location of the bleb relative to PAC was determined. If they overlapped each other, the bleb location was classified as either central or marginal; a central-type bleb had no overlap with a marginal region of PAC, and a marginal-type bleb had at least a partial overlap with a marginal region of PAC.

#### Aneurysmal hemodynamics

The mean WSS at the parent artery (WSS-p) and aneurysmal dome (WSS-d) were computed, including their ratio (normalized WSS, NWSS). The location of the parent artery was chosen immediately upstream of the aneurysm. In aneurysms harboring a bleb, the mean, minimum, and maximum WSS at the bleb including the bleb neck were computed, and the ratio of the maximum WSS at the bleb to that of the whole dome was computed (normalized maximum WSS, NMWSS).

### Statistical analysis

A categorical regression analysis was carried out to identify an independent variable associated with bleb formation among aneurysmal morphology, hemodynamics, and PAC by quantifying nominal, ordinal, and numeric variables using optimal scaling and assigning numerical values to the different categories. The dependent variable was the presence of blebs, and the independent variables were age, sex, shape (dome depth, aspect ratio, dome volume), hemodynamic indices (WSS-p, NWSS), and the presence of PAC. Descriptive analysis of continuous variables was performed, including the calculation of the means, standard deviations, medians, and 99% confidence intervals (99% CI) for all variables. The correlations between the quantitative and qualitative variables were analyzed using Pearson’s or Spearman’s correlation analyses. Accordingly, statistical differences in aneurysmal shape and hemodynamic indices were analyzed between groups using the Kruskal–Wallis test followed by the Steel–Dwass multiple comparison test. Data were analyzed using the IBM Statistical Package for the Social Sciences (SPSS) for Windows version 25 (IBM Corp., Armonk, NY) with the Categories module and R version 4.0.2. Statistical significance was set at *p* < 0.05.

## Results

### Classification of aneurysms

Forty-five aneurysms were classified in terms of the presence of blebs and PAC. Fourteen aneurysms (31.1%) had blebs (group A), and all these aneurysms not only exhibited the presence of PAC, but also the locations of the blebs and PAC were found to match fairly well, which was also confirmed during intraoperative observations for clipped aneurysms. Thirty-one aneurysms (68.9%) had no bleb, of which 13 had PAC (group B) and 18 had no PAC (group C). No patient exhibited a bleb without PAC. Table 2 summarizes these data together with the mean and standard deviations of the ages, shape indices, and hemodynamic indices of the subjects. Details of the data are presented in Supplementary Table 2.

**Table 2:** Aneurysmal shapes and hemodynamic features of the three groups.

Category	Group-A	Group-B	Group-C
Bleb	Yes	No	No
Contact	Yes	Yes	No
Age (y.o.)	64.79 ± 8.65	63.00 ± 12.58	66.78 ± 7.85
Location (Nr.)	14	13	18
MCA	1	10	8
AComA	7	2	6
IC-PC	5	0	0
BA-tip	1	0	3
AChorA	0	1	1
Neck width (mm)	5.71 ± 1.87	3.99 ± 0.85	5.53 ± 9.13
Dome depth (mm)	4.94 ± 2.55	2.83 ± 1.20	4.87 ± 14.77
Aspect ratio	0.87 ± 0.42	0.70 ± 0.33	0.48 ± 0.30
Dome surface area (mm^2^)	118.23 ± 79.69	40.60 ± 23.43	18.77 ± 13.53
Dome volume (mm^3^)	155.64 ± 130.89	34.25 ± 28.12	12.28 ± 12.48
WSS-p	3.07 ± 1.39	5.54 ± 4.46	4.88 ± 3.18
WSS-d	1.24 ± 0.64	3.43 ± 3.35	6.80 ± 5.55
NWSS	0.44 ± 0.24	0.56 ± 0.21	1.34 ± 0.60

Group A: Bleb (Yes), Contact (Yes), Group B; Bleb (No), Contact (Yes); Group C; Bleb (No); Contact (No). NWSS, normalized wall shear stress; WSS, wall shear stress; WSS-p, WSS at the parent artery; WSS-d, WSS at the aneurysmal dome. All data are expressed as mean ± standard deviation.

### Factors associated with bleb formation

Table 3 summarizes the results of a categorical regression analysis of 45 aneurysms to determine a significant factor associated with bleb formation, including aneurysmal shape indices, hemodynamic indices, and the presence of PAC. Since strong positive associations were found between neck width and dome depth (r = 0.752, *p* < 0.001), dome surface area and dome volume (r = 0.997, *p* < 0.001), and WSS-d and NWSS (r = 0.817, *p* < 0.001), these three variables (neck width, dome surface area, WSS-d) were excluded from the independent variables. The categorical regression analysis resulted in F (12, 32) = 4.932, *p* < 0.001, R2 = 0.649, and an adjusted R2 of = 0.517. The presence of PAC was the only significant factor associated with bleb formation (β = 0.476, *p* = 0.038).

**Table 3:** Results of the categorical regression analysis for determining a substantial factor of bleb formation.

Independent variables	Standardized coefficients		Degree of freedom	F-value	*p*-value	Adjusted R^2^
	Beta	SE				
Age (years)	0.101	0.227	1	0.200	0.657	0.517
Sex (0: female/1: male)	0.097	0.118	1	0.669	0.420	
Depth (mm)	0.174	0.419	1	0.172	0.681	
Aspect ratio	-0.221	0.338	3	0.428	0.734	
Volume (mm^3^)	0.536	0.449	2	1.422	0.256	
WSS-p	-0.269	0.250	2	1.158	0.327	
NWSS	0.228	0.234	1	0.955	0.336	
Contact (0: without/1: with contact)	0.476	0.221	1	4.662	0.038	

NWSS, normalized wall shear stress; Contact, perianeurysmal contact; WSS, wall shear stress; WSS-p, WSS at the parent artery.

### Association of aneurysmal shape with hemodynamics

**Figure 1:**
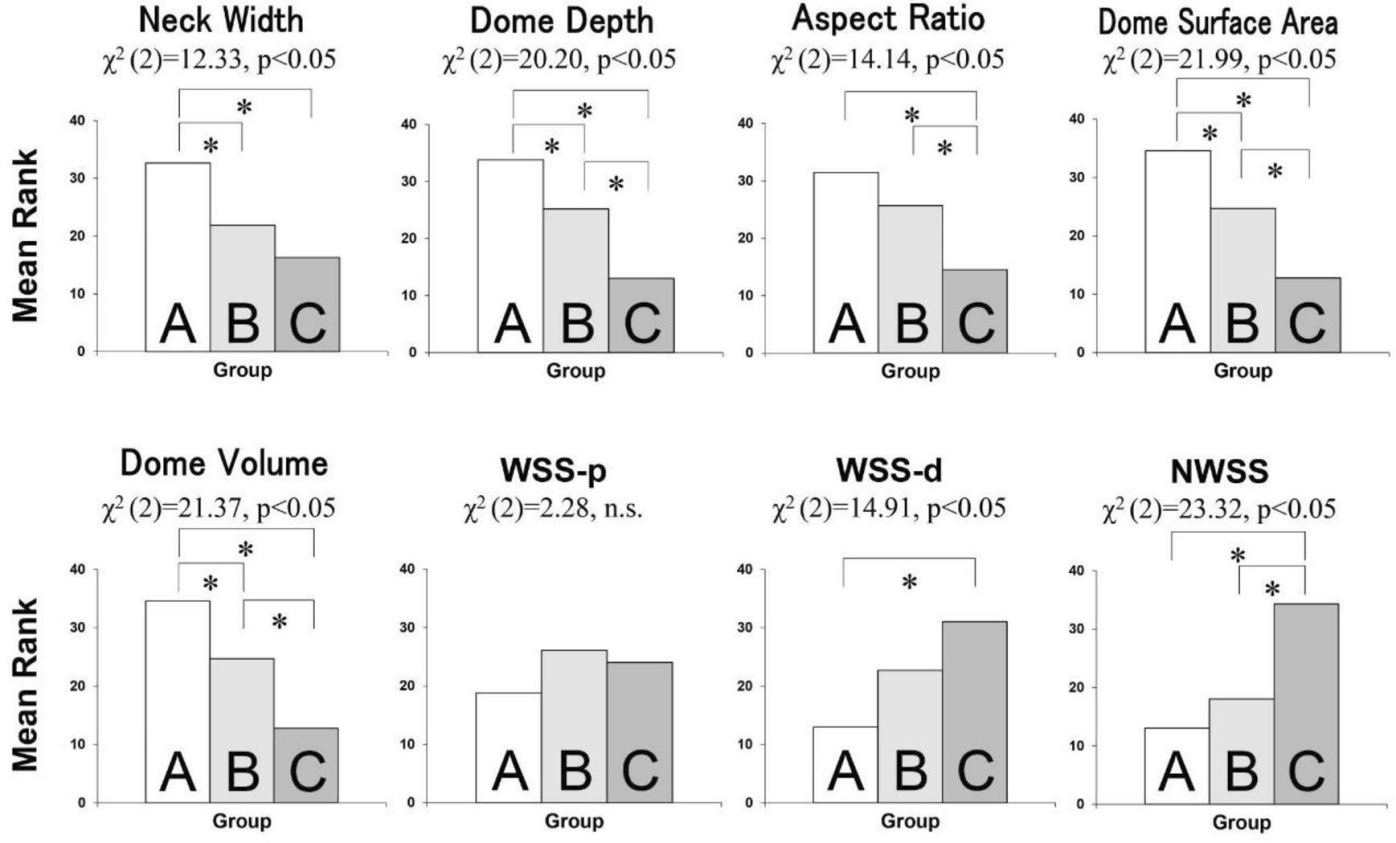
Comparison of aneurysmal shapes and hemodynamic indices between the three groups. (**A**) Group A; (**B**) Group B; (**C**) Group C. * *p* < 0.05.

Aneurysmal shape and hemodynamic indices were compared between the three groups using the Kruskal–Wallis test (Fig. 1). Five shape indices (neck width, dome depth, aspect ratio, dome surface area, and dome volume) and two hemodynamic indices (WSS-d and NWSS) were significantly different: neck width: χ2(2) = 12.33, *p* < 0.05; dome depth: χ2 (2) = 20.20, *p* < 0.05; aspect ratio: χ2 (2) = 14.14, *p* < 0.05; dome surface area: χ2 (2) = 21.99, *p* < 0.05; dome volume: χ2 (2) = 21.37, *p* < 0.05; WSS-p: χ2 (2) = 2.28, n. s.; WSS-d: χ2 (2) = 14.91, *p* < 0.05; and NWSS: χ2 (2) = 23.32, *p* < 0.05). Dome depth, dome surface area, and dome volume were significantly higher in groups A, B, and C in series (*p* < 0.05, Steel–Dwass multiple comparison test). Aneurysmal wall shear stress (WSS-d, NWSS) tended to be lowest in group A, followed by groups B and C in series, but the difference between groups A and B was not statistically significant.

### Association of blebs with hemodynamics

**Figure 2:**
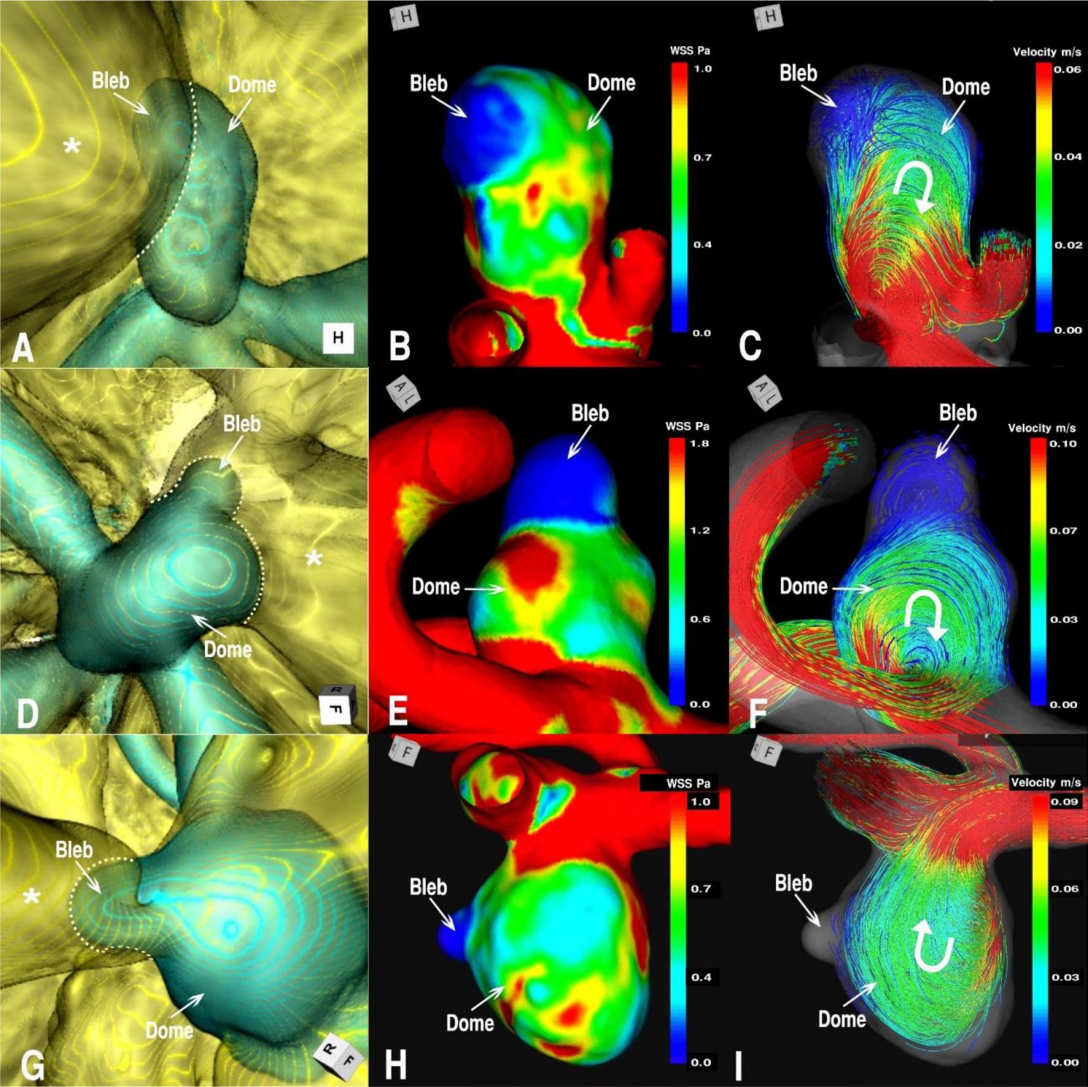
Relationship between a bleb and PAC with hemodynamics (WSS and streamlines) in group A. Blebs at the inflow region in AComA case 3 (**A**–**C**), at the intermediate region in AComA case 8 (**D**–**F**), and at the outflow region in case 10 of the left IC-PC (**G**–**I**) aneurysms are depicted. (**A, D, G**) 3D multifusion images of CTA and MRC visualizing the blebs in contact with the adjacent rectal gyri of the frontal lobe (*) in A and D, and with the oculomotor nerve (*) in G. The dotted lines represent the borders of contact with the brain or cranial nerves. Note that the blebs are located in the marginal region of the contact area. (**B, E, H**) Distribution of the mean WSS over the bleb and whole dome, showing relatively low WSS at the bleb. (**C, F, I**) Intra-aneurysmal streamlines in relation to the bleb, showing a bleb at the inflow (C), intermediate (F), and outflow (I) locations. The curved arrows indicate the direction of the streamlines.

In group A, the association between bleb formation and aneurysmal hemodynamics was investigated. Table 4 summarizes the mean, minimum, and maximum WSS of the blebs, including the bleb necks and the whole domes together with the ratio of the maximum WSS of the bleb to that of the whole dome, referred to as the NMWSS. The NMWSS is an indicator of the elevation of the WSS at the bleb in each aneurysm. The NMWSS ranged from 0.03% to 0.36%, with a mean of 0.17 ± 0.10 (mean ± SD). In other words, the maximum WSS at the bleb was only 17% of the maximum WSS in the entire dome. By analyzing streamlines, the bleb locations were investigated to determine whether they were located in the inflow, intermediate, or outflow regions. In our datasets, we were unable to find a tendency for an association between bleb location and the flow regions. Figure 2 shows representative cases with blebs at inflow, intermediate, and outflow regions.

**Table 4:** Hemodynamic details of WSS and the relationship between blebs and PAC in group A.

Case No	Location of aneurysms	WSS of Bleb mean (Min–Max) Pa	WSS of whole dome mean (Min–Max) Pa	NMWSS	Contact	Location of Bleb at contact area	Outcome
1	Rt ICPC	0.33 (0.10–1.76)	0.60 (0.10–10.0)	0.18	Medial temporal lobe	Marginal region	Clipped
2	AComA	0.93 (0.21–2.01)	1.12 (0.21–8.53)	0.24	Frontal lobe	Marginal region	F/U
3	AComA	0.23 (0.21–0.27)	1.54 (0.21–5.11)	0.05	Frontal lobe	Marginal region	Clipped
4	AComA	1.29 (0.28–3.39)	1.70 (0.01–15.45)	0.22	Frontal lobe	Marginal region	Coiled
5	Rt ICPC	0.16 (0.03–1.42)	1.07 (0.03–7.12)	0.20	Medial temporal lobe	Marginal region	F/U
6	Rt ICPC	1.14 (0.15–3.12)	2.81 (0.15–14.60)	0.21	Medial temporal lobe	Marginal region	Coiled
7	Lt ICPC	0.12 (0.03–0.26)	1.80 (0.03–8.46)	0.03	Medial temporal lobe	Central region	Clipped
8	AComA	0.31 (0.07–1.44)	0.98 (0.04–5.93)	0.24	Frontal lobe	Marginal region	Clipped
9	Lt MCA	0.03 (0.01–0.11)	0.26 (0.01–3.70)	0.03	Temporal lobe	Marginal region	F/U
10	Lt ICPC	0.06 (0.01–0.24)	0.45 (0.01–5.54)	0.04	Oculomotor nerve	Marginal region	Clipped
11	AComA	0.80 (0.23–1.49)	1.58 (0.23–7.01)	0.21	Optic chiasma	Marginal region	Clipped
12	AComA	0.30 (0.03–1.46)	0.98 (0.03–5.83)	0.25	Optic chiasma	Marginal region	Clipped
13	AComA	0.43 (0.11–1.29)	1.34 (0.11–15.32)	0.08	Frontal lobe	Marginal region	F/U
14	BA-tip	0.34 (0.07–1.61)	1.12 (0.07–4.48)	0.36	Diencepharon	Marginal region	SAH
Overall		0.11 (0.03–3.39)	0.45 (0.01–15.45)	0.17 ± 0.10 (*n* = 14)			

NMWSS, normalized maximum wall shear stress; SAH, subarachnoid hemorrhage.

### Association of blebs with PAC

Table 4 summarizes the association of bleb formation with PAC (group A). First, all of these blebs coincided with the location of the PAC. Furthermore, most blebs were located in the marginal region of the PAC (93%). The three cases shown in Fig. 2 illustrate these blebs. Tissues under contact included the brain parenchyma and the cranial nerves.

## Discussion

The rupture risk of UIAs during follow-up is often diagnosed based on their morphology, such as their size, aspect ratio, and bleb formation^3,4,6^. Blebs may be formed due to a focal increase in wall vulnerability, but the mechanism of bleb formation in UIAs remains to be understood. The present study is the first to examine the spatial characteristics of bleb location in UIAs in terms of both PAC and intra-aneurysmal hemodynamics.

The main result of this study was the discovery that aneurysms harboring blebs not only exhibited the presence of PAC, but also the location of the blebs matched that of the PAC. In addition, PAC was an independent variable significantly associated with bleb formation, as opposed to aneurysmal shape and hemodynamic indices. We also found that aneurysms harboring blebs were significantly larger than those without blebs. In particular, a comparison of group A (bleb (+), PAC (+)) with group B (bleb (−), PAC (+)) suggested that bleb formation may be associated with the establishment of PAC during aneurysmal growth.

The pathophysiological mechanism of intracranial aneurysms has been studied using human pathological specimens^19–22^ and animal models^23–26^. Flow-induced, inflammation-mediated biological reactions that are thought to be involved in the formation and progression of intracranial aneurysms^27^. To the best of our knowledge, however, there has been no reports that investigated the pathology of bleb. Since a bleb is a focal secondary bulge of aneurysmal wall, the underlying mechanism may be associated with a focal increase of wall vulnerability.

As previously described, the formation and progression of intracranial aneurysms may be linked to aberrant hemodynamics^19,27^. As for bleb formation, two schools of thought exist: one supports strong inflow jets and high WSS^11–13^ and the other supports low WSS and a high shear stress gradient^14,15^. In our study, we first classified the bleb locations into inflow, intermediate, and outflow regions; however, we were unable to find a specific tendency between the bleb location and flow region. Then, we focused on the WSS at the bleb neck, which was assumed to reflect the WSS at the initiation of bleb. The amount of WSS was only 17% of that of the entire dome. This finding suggests that bleb formation may not be associated with an elevation of WSS. Since the present study treated limited sample sizes, understanding the association between WSS and bleb formation may need larger samples.

Another pathway to increase the vulnerability of aneurysmal walls may be involved. The establishment of PAC may focally constrain the deformation of aneurysmal wall in a mechanical point of view, resulting in a state of stress concentration by which the wall vulnerability is focally accelerated. Vascular wall stress (VWS) is a mechanical stress that acts on the inside of the vascular wall, which is linked to blood pressure, and is different from WSS, which acts on the inner surface of the vascular wall due to fluid viscosity and has no association with blood pressure. Although there were a number of studies that reported on the biomechanics of intracranial aneurysms, most studies focused on WSS only and did not consider the effect of VWS, despite the fact that the VWS (often termed ‘stretch’) is deeply associated with the conditions of smooth muscle cells (SMCs)^28^. Kataoka et al.^29^ speculated the VWS may be associated with the initiation of aneurysm, and WSS may not be, as opposed to the conventional theory that related the aneurysmal initiation with the elevation of WSS. Seshaiyer and Humphrey^30^ investigated VWS together with the effect of mechanical constraint using finite element analysis. They demonstrated that the wall constraint decreased the VWS, but there was an exceptional location, or a site near the contact, where the VWS significantly increased (stress concentration). Although their simulation did not include the effect of biological reactions, a concentrated VWS may cause SMC death. Indeed, various reports have shown an association between VWS and SMC apoptosis^28^. Furthermore, SMC death is known to be involved in ruptured intracranial aneurysms in human specimens^22^. These insights led us to hypothesize that the emergence of PAC may yield a concentrated VWS, cause cell death of SMCs, and result in bleb formation due to the increase in wall vulnerability.

## Limitation

This study had several limitations. The sample size was limited, particularly in group A. Future studies should increase the number of aneurysms. Furthermore, future work should include a prospective study to investigate bleb formation over time. The presence of PAC was judged using 3D multifusion imaging, which has a certain uncertainty due to imaging and thresholding. Therefore, in this study, we confirmed the presence of PAC with intraoperative observations for clipped aneurysms. CFD needed various assumptions, such as the blood properties and boundary conditions. Although these uncertainties existed, our interpretation were believed to be unassociated with these uncertainties.

## Conclusion

This study is the first to examine the spatial environment of bleb location in UIAs in terms of both PAC and intra-aneurysmal hemodynamics. The data demonstrated that bleb formation in UIAs was associated with the establishment of PAC during their growth, which may have more detrimental effects on bleb formation than hemodynamics.

## Supplementary Information

Supplementary Information.

## Abbreviations

3D: 3-Dimensional
CFD: Computational fluid dynamics
CTA: Computed tomographic angiography
MRA: Magnetic resonance angiography
MRC: Magnetic resonance cisternography
MRI: Magnetic resonance imaging
PAC: Peri-aneurysmal contact
VWS: Vascular wall stress
WSS: Wall shear stress
UIA: Unruptured intracranial aneurysm
