# Supplementary material for: Association of bleb formation with peri-aneurysmal contact in unruptured intracranial aneurysms
Source: Sci Rep. 2022 Apr 12;12:6075. doi: 10.1038/s41598-022-10064-8 (PMC9005541; doi:10.1038/s41598-022-10064-8)
Supplement: Supplementary file 1 — Supplementary Information. [file 41598_2022_10064_MOESM1_ESM.pdf]

Supplemental raw data of TABLE 1.

Aneurysmal shapes and hemodynamic features of three groups.

Group-A; Bleb (Yes), Contact (Yes), Group-B; Bleb (No), Contact (Yes), Group-C; Bleb (No), Contact (No).

Group	No	Bleb	Contact	Age/Sex	Location of Aneurysms	Width (mm)	Depth (mm)	Aspect Ratio	Surface (mm2)	Volume (mm3)	WSS-p	WSS-d	NWSS
A	1	Yes	Yes	68/F	Rt ICPC	7.02	2.91	0.42	67.53	66.84	5.20	0.60	0.12
	2	Yes	Yes	63/F	AComA	6.11	5.11	0.84	112.74	146.99	2.70	1.12	0.41
	3	Yes	Yes	72/F	AComA	4.97	3.13	0.63	48.95	42.01	2.68	1.54	0.57
	4	Yes	Yes	58/M	AComA	6.55	9.09	1.39	222.15	285.83	6.89	1.70	0.25
	5	Yes	Yes	75/F	Rt ICPC	8.38	7.43	0.89	200.31	305.37	2.81	1.07	0.38
	6	Yes	Yes	49/F	Rt ICPC	2.61	1.21	0.46	9.91	4.29	3.07	2.81	0.92
	7	Yes	Yes	58/M	Lt ICPC	3.25	1.84	0.56	21.25	11.86	2.52	1.80	0.71
	8	Yes	Yes	74/F	AComA	4.88	5.73	1.17	88.14	86.87	2.38	0.98	0.41
	9	Yes	Yes	68/F	Lt MCA	4.01	5.84	1.46	102.13	107.58	2.34	0.26	0.11
	10	Yes	Yes	66/M	Rt ICPC	5.65	9.90	1.75	270.13	439.66	1.39	0.45	0.32
	11	Yes	Yes	75/F	AComA	4.44	2.98	0.66	41.92	35.52	1.94	1.58	0.81
	12	Yes	Yes	50/M	AComA	6.52	4.68	0.72	122.28	151.86	2.66	0.98	0.37
	13	Yes	Yes	61/F	AComA	6.24	4.91	0.79	184.03	243.38	3.18	1.34	0.42
	14	Yes	Yes	70/F	BA-tip	9.36	4.36	0.47	163.78	250.87	3.27	1.12	0.34
	Ave±SD	-	-	64.79±8.65	-	5.71±1.87	4.94±2.55	0.87±0.42	118.23±79.69	155.64±130.89	3.07±1.39	1.24±0.64	0.44±0.24
	Mdn	-	-	67.00	-	5.88	5.88	0.75	107.43	127.29	2.69	1.12	0.40

	**99%CI**	**-**	**-**	**58.83-70.74**	-	4.43-7.00	3.18-6.69	0.59-1.16	63.37-173.09	65.53-245.75	2.12-4.03	0.80-1.68	0.27-0.60
	**15**	**No**	**Yes**	**77/F**	AComA	4.20	3.00	0.70	45.20	36.30	2.86	1.02	0.36
	**16**	**No**	**Yes**	**53/M**	AComA	3.70	2.70	0.40	32.60	22.20	3.30	2.10	0.64
	**17**	**No**	**Yes**	**39/F**	Rt AChor	3.20	1.20	0.40	12.60	5.90	3.20	2.60	0.81
	**18**	**No**	**Yes**	**49/F**	Rt MCA	4.30	2.80	0.70	41.93	32..6	2.60	1.70	0.65
	**19**	**No**	**Yes**	**67/F**	Rt MCA	3.50	3.40	1.00	35.65	25.70	3.20	2.00	0.63
	**20**	**No**	**Yes**	**78/F**	Rt MCA	2.90	3.10	1.10	37.77	25.60	11.70	9.70	0.83
	**21**	**No**	**Yes**	**63/M**	Rt MCA	6.30	2.60	0.40	50.10	48.60	4.50	3.40	0.76
**B**	**22**	**No**	**Yes**	**73/F**	Rt MCA	3.30	1.20	0.40	13.53	7.30	8.80	3.90	0.44
	**23**	**No**	**Yes**	**78/F**	Lt MCA	4.01	5.84	1.46	102.13	107.58	2.30	0.30	0.13
	**24**	**No**	**Yes**	**67/F**	Lt MCA	4.50	2.70	0.60	43.20	42.30	13.50	10.40	0.77
	**25**	**No**	**Yes**	**60/M**	Lt MCA	4.00	2.10	0.50	24.20	16.00	1.30	0.70	0.54
	**26**	**No**	**Yes**	**48/F**	Lt MCA	3.50	2.20	0.60	24.89	15.80	2.00	0.60	0.30
	**27**	**No**	**Yes**	**67/F**	Lt MCA	4.40	4.00	0.90	64.06	57.70	12.80	6.20	0.48
	**Ave±SD**	**-**	**-**	**63.00±12.58**	-	3.99±0.85	2.83±1.20	0.70±0.33	40.60±23.43	34.25±28.12	5.54±4.46	3.43±3.35	0.56±0.21
	**Mdn**	**-**	**-**	**67.00**	-	4.00	2.70	0.60	37.77	25.65	3.20	2.10	0.63
	**99%CI**	**-**	**-**	**54.01-71.99**	-	3.38-4.60	1.98-3.69	0.47-0.94	23.87-57.34	14.16-54.34	2.36-8.73	1.04-5.82	0.41-0.72
	**28**	**No**	**No**	**77/M**	AComA	3.60	1.40	0.40	15.80	9.30	7.00	7.50	1.07
	**29**	**No**	**No**	**53/M**	AComA	4.00	1.60	0.40	20.20	11.60	2.00	2.30	1.15
	**30**	**No**	**No**	**66/M**	AComA	4.80	1.70	0.40	26.40	17.40	1.80	2.90	1.61
	**31**	**No**	**No**	**71/F**	AComA	2.30	1.00	0.40	7.23	3.10	11.60	10.30	0.89
	**32**	**No**	**No**	**47/M**	AComA	2.20	0.40	0.20	4.21	2.00	3.80	5.40	1.42
	**33**	**No**	**No**	**65/F**	AComA	2.90	0.90	0.30	8.80	3.40	7.10	17.70	2.49

	**34**	**No**	**No**	**73/F**	Lt AChor	2.80	0.80	0.30	7.90	3.10	7.90	19.40	2.46
	**35**	**No**	**No**	**67/F**	Rt MCA	3.40	2.20	0.70	25.20	16.60	10.30	9.40	0.91
**C**	**36**	**No**	**No**	**61/F**	Rt MCA	4.10	1.60	0.40	21.17	12.80	2.60	4.10	1.58
	**37**	**No**	**No**	**61/F**	Rt MCA	2.60	1.00	0.40	8.21	4.30	5.90	7.70	1.31
	**38**	**No**	**No**	**74/F**	Rt MCA	3.70	0.70	0.20	13.62	6.17	1.70	2.04	1.20
	**39**	**No**	**No**	**68/F**	Lt MCA	3.20	2.30	0.70	23.50	15.30	3.70	6.90	1.86
	**40**	**No**	**No**	**72/M**	Lt MCA	3.30	1.20	0.40	13.00	9.10	7.70	9.00	1.17
	**41**	**No**	**No**	**71/F**	Lt MCA	2.70	1.00	0.40	8.74	5.10	6.30	12.50	1.98
	**42**	**No**	**No**	**74/F**	Lt MCA	3.30	1.80	0.60	17.80	10.40	2.20	1.00	0.45
	**43**	**No**	**No**	**72/F**	BA-tip	5.00	1.60	0.30	28.07	18.60	1.90	1.60	0.84
	**44**	**No**	**No**	**60/F**	BA-tip	42.00	64.00	1.52	63.72	57.18	3.10	0.80	0.26
	**45**	**No**	**No**	**70/M**	BA-tip	3.70	2.40	0.60	24.31	15.60	1.30	1.80	1.38
	**Ave±SD**	**-**	**-**	**66.78±7.85**	-	5.53±9.13	4.87±14.77	0.48±0.30	18.77±13.53	12.28±12.48	4.88±3.18	6.80±5.55	1.34±0.60
	**Mdn**	**-**	**-**	**69.00**	-	3.35	1.50	0.40	16.80	9.85	3.75	6.15	1.25
	**99%CI**	**-**	**-**	**62.01-71.54**	-	-0.01-11.08	-4.10-13.83	0.30-0.66	10.56-26.99	4.70-19.86	2.95-6.81	3.43-10.17	0.97-1.70
	**Overall**												
	**Ave±SD**	**-**	**-**	**65.07±9.57**	-	5.14±5.83	4.30±9.35	0.67±0.38	56.02±62.98	63.19±96.69	4.51±3.31	4.10±4.56	0.83±0.59
	**Mdn**	**-**	**-**	**67.00**	-	4.00	2.40	0.60	28.07	18.60	3.10	2.00	0.71
	**99%CI**	**-**	**-**	**61.39-68.74**	-	2.90-7.38	0.71-7.89	0.52-0.81	31.84-80.20	26.06-100.32	3.24-5.78	2.34-5.85	0.61-1.06

AChorA=anterior choroidal artery; AComA= anterior communicating artery; Ave=mean; BA-tip=the tip of the basilar artery; CI=confidence interval; IC-PC=internal carotid-posterior communicating artery; Lt= left; MCA=middle cerebral artery;

Mdn=median; NWSS=normalized wall shear stress; Rt= right; SD=standard deviation; WSS= wall shear stress; WSS-p=WSS at parent artery; WSS-d=WSS at aneurysmal dome; All data=mean ± standard deviation.
